# Cerebral Pathology Based upon the Examination of 411 Cases. Specific Gravity of the Brain in Cases of Insanity

**Published:** 1855-10-01

**Authors:** David Skae

**Affiliations:** Physician to the Royal Edinburgh Asylum for the Insane, &c., &c.


### CEREBRAL PATHOLOGY BASED UPON THE EXAMINATION OF 411 CASES. SPECIFIC GRAVITY OF THE BRAIN IN CASES OF INSANITY.

BY DAVID SKAE, M.D.,

*Physician to the Royal Edinburgh Asylum for the Insane, &c., &c.*

We are glad of an opportunity of transferring to our pages the following valuable communication of Dr. Skae, published in the Annual Report of the *Royal Edinburgh Asylum* for 1854. It refers to a subject of deep and increasing interest. We regret that want of space compels us to omit the tables illustrative of Dr. Skae's experiments in connexion with the comparative weights of the brain in the various forms of mental disease, as well as other tabular matter, showing the specific gravity of the grey and white substance of the brain in the sane and the insane. We, however, give all the results at which he has arrived in relation to both branches of inquiry.

*Post-mortem* examinations were permitted in 41 cases. They were made with much care, and the following are the results:—

Of those examined, 3 had been cases of acute mania, 15 of dementia, 5 of dementia with epilepsy, 3 of melancholia, 9 of monomania, and 6 of general paralysis.

*Calvarium was of unusual thickness* in 6 cases; 1 of acute dementia, 1 of dementia, 3 of dementia with epilepsy, and 1 of melancholia.

*Calvarium was thinner than usual* in 9 cases; 2 of melancholia, 1 of dementia, 4 of monomania, and 2 of general paralysis.

*Diploe was absent* in 2 cases of monomania.

*Abnormal adhesion of dura mater to calvarium* existed in 4 cases; 1 of dementia with epilepsy, 2 of monomania, and 1 of general paralysis.

*Pus in the cranial sinuses* was found in 2 cases; 1 of dementia with epilepsy, and 1 of acute dementia; in the latter case, *pus* was also found in the internal ear, and *sero-purulent effusion* beneath the arachnoid, and in the lateral ventricles.

*Increased thickness of dura mater* was found in 3 cases; 1 of acute mania, 1 of acute dementia, and 1 of dementia with epilepsy.

*Thinness of dura mater* was noticed in five cases; 2 of dementia, and 3 of monomania.

*Ossific deposit in falx cerebri* existed in 1 case of monomania.

*Abnormal adhesion of arachnoid to dura mater* was found in 4 cases; 1 of acute dementia, 1 of dementia with epilepsy, 1 of monomania, and 1 of general paralysis.

*Serous effusion into sac of arachnoid* existed in 22 cases; 1 of acute dementia, 8 of dementia, 2 of dementia with epilepsy, 5 of monomania, and 6 of general paralysis.

*Extravasation of blood into sac of arachnoid* was found in 2 cases of general paralysis.

*Organised lymph in sac of arachnoid* was found in 1 case of general paralysis.

*Opacity and thickening of arachnoid* was found in 26 cases; 1 of acute mania, 2 of acute dementia, 7 of dementia, 6 of dementia with epilepsy, 2 of melancholia, 4 of monomania, and 4 of general paralysis.

*Granular deposit in arachnoid over general surface* was noticed in 2 cases of general paralysis.

*Congestion of membranes* was noticed in 12 cases; 1 of acute mania, 3 of dementia, 1 of acute dementia, 2 of dementia with epilepsy, 1 of monomania, and 4 of general paralysis.

*Sub-arachnoid serous effusion* was found in 22 cases; 1 of acute mania, 1 of acute dementia, 6 of dementia, 2 of dementia with epilepsy, 6 of monomania, and 6 of general paralysis.

*Sub-arachnoid sero-sanguinolent effusion* occurred in 1 case of dementia with epilepsy.

*Adhesion of membranes to cortical substance* was found in 3 cases of general paralysis.

*Paleness of the grey matter* was noticed in 17 cases; 2 of melancholia, 6 of dementia, 1 of acute dementia, 6 of monomania, and 2 of general paralysis.

*Grey matter* was of a *dark tint* in 3 cases; 1 of dementia with epilepsy, 1 of dementia, and 1 of melancholia.

*Grey matter had a violaceous tinge* in 5 cases; 1 of acute mania, 1 of dementia with epilepsy, 1 of monomania, and 2 of general paralysis.

*Grey matter had a yellow tint* in 2 cases; 1 of acute mania, and 1 of general paralysis.

*Grey matter was softened* in 2 cases; 1 of dementia, and 1 of general paralysis.

*Grey matter presented limited yellow softening* in 3 cases; 2 of dementia with epilepsy, and 1 of general paralysis.

*White matter was softened* in 2 cases: 1 of dementia, and 1 of general paralysis.

*Punctæ vasculosæ* were very numerous in 15 cases; 1 of acute dementia, 2 of dementia, 5 of dementia with epilepsy, 1 of melancholia, 2 of monomania, and 4 of general paralysis.

*Apoplectic effusion* was found in 1 case of monomania.

*Serous effusion into lateral ventricles* was found in 16 cases; 2 of acute mania, 1 of melancholia, 5 of dementia, 2 of dementia with epilepsy, 2 of monomania, and 4 of general paralysis.

*Sero-sanguinolent effusion into lateral ventricles* occurred in 1 case of acute dementia.

*Granular deposit in membrane of lateral ventricles* was found in 3 cases; 1 of dementia with epilepsy, and 2 of general paralysis.

*Cystic bodies in choroid plexuses* occurred in 9 cases; 1 of acute mania, 1 of dementia with epilepsy, 4 of monomania, and 3 of general paralysis.

*Pineal body* was *unusually large* in 4 cases of dementia.

*Veins of Galen were much dilated and clogged with decolorized clots* in 1 case of dementia with epilepsy.

The brain was carefully weighed in every case, and the weights, with those of other viscera, together with the specific gravity of the grey and white matter of the brain are recorded.

The general conclusions to be derived from the preceding abstract are, that, in a large proportion of the deaths, well-marked appearances were found indicating increased vascular action, or slow inflammatory processes affecting the membranes of the brain, and producing thickening and serous effusions. These appearances were most constant and most distinct in the deaths from general paralysis, where there was also found a peculiar kind of *softening of the grey substance* of the brain, and on examining this part of the brain under the microscope, the cells of which it is principally composed appeared to be enlarged and altered in form. In these cases, *adhesion* of the membranes to the surface of the convolutions was present in all instances where there was not such a quantity of serous effusion interposed as to prevent it; in stripping off the membranes, small layers of grey matter were stripped off with them, particularly on the lateral surfaces of the hemispheres.

With the exception of the morbid changes last mentioned, which are limited to cases of general paralysis, all the other morbid appearances are found very frequently in the brains of persons who have died of other diseases, and without any mental affection. And, on the other hand, cases are not unfrequently to be met with in the dead-house of the Asylum, of patients who had exhibited, during life—for many months or even years—all the symptoms of raving madness, and in whose brains the morbid appearances described are altogether wanting, and in which the brain could not be distinguished from that of one who had died in all the vigour of a sound mind.

From these facts it must be inferred that the morbid appearances described, although the very frequent concomitants of insanity, do not constitute the pathological conditions by which the symptoms of mental derangement are produced; and that we are, in fact, ignorant of the true pathology of this disease.

There are many considerations which may well lead us to doubt whether any other morbid changes in the structure of the brain may ever be discoverable in insanity. The analogy of the symptoms to those produced by poisons which are known to enter the blood, the suddenness of the invasion in some cases, and the suddenness of the cure in others, even occasionally of long standing, the remarkable remissions, and at times the temporary restoration for a few hours or moments, to perfect sanity in persons long plunged in profound dementia, or labouring under a chronic and protracted mania, are facts which lead to the reflection that insanity may be a disease of the blood, or may depend upon a peculiar orgasm of the nervous centres,—upon conditions not to be discovered in the brain after death.

Pathological investigations, however, for changes in the nervous substance itself, are not to be abandoned until we have exhausted all our means of observation; and it occurred to me, accordingly, some years ago, to commence a series of experiments by collecting the *weights* of the brain, cerebrum and cerebellum,—by measuring the *breadths* of the *grey matter*,—by endeavouring to determine the degree of *hardness or softness* of the cerebral substance, by ascertaining by what length of a column of water it could be broken up,—and, more lately, by taking the *specific gravity* of the grey and white substance of different parts of the brain.

In an hospital for the insane, where the deaths are much fewer than in a large general hospital, it takes some years before a sufficient number of data can be accumulated to warrant any general inferences; and I feel that the data which I possess, although the accumulation of some years, are still rather meagre. I am induced, however, to record them, as offering a few very interesting suggestions, which may lead others to follow similar methods of observation, and thus facilitate the collection of a larger number of facts.

My measurements of the grey matter, and experiments on the hardness and softness of the cerebral substance, I do not propose to detail at present, as I have no similar observations on the brains of persons dying sane with which to compare them. I shall confine myself, therefore, to the *weight* and *specific gravity* of the brain, and to such general deductions only from those observations as the number of facts seem to warrant. I shall leave for another opportunity several comparisons and inquiries which might be instituted through these methods, inasmuch as the data seem to me too few to justify speculations which relate to more special questions.

I have compared my observations on the weights of the brain with those collected in the Royal Infirmary of Edinburgh by the late Dr. John Reid and Dr. Peacock, and the experiments on its specific gravity with those published by Dr. Sankey in the "British and Foreign Medico-Chirurgical Review," for January, 1853 (vol. xi., p. 240), and made in the Royal London Fever Hospital. The weights used were avoirdupois.

The observations made of the weights of the brain comprise 199 cases, of which 98 were males and 101 females. The tables exhibit the weights of the heaviest and lightest encephalon, cerebrum, and cerebellum (with the pons and medulla), and their average weights at different periods of life. The figures are arranged in parallel columns with those of the corresponding results from the tables of Drs. Reid and Peacock.

On comparing the columns showing the heaviest brains in the sane and insane *males*, it will be seen that in only one instance did the encephalon exceed 60 ounces in the insane, while in several of the sane it amounted to 62 ounces and upwards, the heaviest brain in the one series being 60 oz. 8 dr., and the heaviest in the other being 62 oz. 12 dr. It will also be seen that, with two exceptions, the superiority in weight was among the brains of the sane in each of the quinquennial or decennial periods into which they are classified. Whether this justifies the inference that persons having large brains are less frequently the subject of mental derangement than others, I shall not venture to say; but the fact is a striking one when contrasted with the results derived from a comparison of the average weight of the entire number of cases, where it appears that the average weight is increased in persons dying insane. The average weight in the insane from fifteen to ninety years of age being 50 oz. 2 dr., and in the sane 49 oz. 14 dr.

Different results are derived from a comparison of the weights of the brains of the *females;* here, in one exceptional case, the brain weighed 61½ ounces in a female who died insane, but in a majority of cases the greatest weight was on the side of the sane. On taking the average of all the cases, the weight of the brain in the insane was 44 oz. only, and in the sane 44 oz. 5 dr.

The comparison, however, establishes, I think, a strong presumption that the absolute weight of the brain is increased in the insane, when we reflect that in many cases of insanity, the absolute *size* of the brain must be materially diminished by the large quantity of serous effusion found in the ventricles, arachnoid sac, and sub-arachnoid tissue.

This increase in weight, however, appears to depend chiefly upon an increase in the weight of the *cerebellum;* for, on comparing the weight of the cerebra in the two series of cases, it will be found that the difference is inconsiderable, and indeed in the case of the females that it is in favour of the sane. On comparing, however, the columns showing the weight of the cerebellum (with the pons and medulla) in the two classes, it will be found that there is almost a uniform preponderance in the weights of those of the insane. The average weight in all the cases of the insane males exceeds that of the sane by 4 drachms, and in the females by 5 drachms.

In a Table the ratio between the cerebellum (with the pons and medulla) and the cerebrum is given at the different ages distinguished. Here the same fact is very clearly brought out; in almost every instance the cerebellum in the insane being heavier in relation to the cerebrum than it is in the sane. From the average of the entire number, the cerebellum was found in the males to be as 1 to 6·748 in the insane, and as 1 to 7·06 in the sane; and in the females as 1 to 6·64 in the insane, and only 1 to 7 in the sane. The cerebellum therefore is considerably heavier in relation to the cerebrum in the insane.

This mode of estimating the results appears to me to be free from the source of fallacy arising from the small number of cases compared; when we compare the absolute weights together, as here, on examining all the cases separately, we find, in almost every instance, the cerebellum of each brain heavier in relation to its own cerebrum in the insane than in the sane.

Further inquiries, and a larger number of data, may modify these results; but, I think, from those which I have thus collected, it may be regarded as highly probable that there is *an increase in the weight of the cerebellum in persons dying insane.*

In mania, the average weight of the encephalon in males was 53 oz. $13\frac{1}{7}$ dr.; in monomania, 52 oz. $1\frac{15}{17}$ dr.; in dementia, 49 oz. $11\frac{3}{13}$ dr.; and in general paralysis 46 oz. $6\frac{12}{13}$ dr.; the weight being greatest in mania, and least in general paralysis. In comparing the average weights of the cerebella, &c., however, in the same series, while they are found to follow the same decrease from mania, the highest, through monomania to dementia, the weights being respectively 6 oz. 12 dr., 6 oz. $11\frac{9}{17}$ dr., and 6 oz. $9\frac{2}{13}$ dr.; the cases of general paralysis present the highest average, the average weight being 6 oz. $13\frac{1}{13}$ dr. From the fact that in general paralysis the morbid appearances are most constant and most distinctly marked, this result might perhaps be anticipated, if we assume what I have endeavoured to show, that the relative weight of the cerebellum to the cerebrum is increased by insanity, and that the cerebellum is the organ principally affected. This increase, too, would appear from a review of the history of the case to bear a constant relation to the form of the disease, and to be greatest in more protracted and gravest cases. On comparing the average weight of the cerebellum to that of the cerebrum in the cases classified as above, it was found in males to be as 1 to 6·9735, 6·7549, 6·5625, and 6·2511, in mania, monomania, dementia, and general paralysis respectively. Excepting mania, the same gradual increase in the relative weight of the cerebellum was found to obtain in the females, the cerebellum being to the cerebrum in them as 1 to 6·5338, 6·412, 6·5943, and 6·0435 in same class of cases respectively.

It appears, therefore, that in cases of comparatively short duration, there is the smallest amount of increase in the relative weight of the cerebellum, and that in general paralysis the greatest increase takes place.

In reflecting upon these results, the impaired control over the voluntary movements, which forms so marked a feature of general paralysis, and the very constant increase of the relative weight of the cerebellum in this disease, must at once be viewed as very interesting and important facts in connexion with the functions ascribed to the cerebellum by Fleurens and other physiologists.

May it not be asked—supposing it to be established by an extended series of observations, that there is uniformly an increase in the relative weight (and specific gravity, as we shall presently see,) of the cerebellum as compared with the cerebrum in all forms of insanity—whether it may not be inferred that the cerebellum is the organ through which we exercise self-control,—control over the volitions and successions of our thoughts, as well as over the voluntary muscular movements;—a perturbed volition or a loss of self-control, being of all other symptoms the most essential characteristic and pathognomonic feature of insanity?

The specific gravity of the *grey* and *white* substance of the brain was taken in 80 cases, of which 39 were males and 41 females.

The specific gravity in the cases of insanity was almost uniformly higher, and this observation applies to both the *grey* and *white* matter.

In Dr. Sankey's cases, the lowest specific gravity of the grey matter was 1028, in the Asylum ones the lowest was 1030; the highest in Dr. Sankey's cases was 1046, in mine 1049; while the average specific gravity in all the cases of both sexes was in the former 1034, and in the latter 1038, showing an increase in the specific gravity in the cases of insanity.

The lowest specific gravity of the medullary substance in the healthy brains was 1032, in the diseased 1032; the highest in the healthy was 1048, in the diseased 1053; the mean of all the cases was 1·0411 in the sane, and 1·0429 in the insane, showing an increase in the specific gravity of the *white* matter of the brain in cases of insanity.

These results are corroborative of those obtained by Dr. Sankey in his observations at the London Fever Hospital, where in all the cases complicated with cerebral symptoms of a grave character preceding death, such as convulsions, strabismus, paralysis, and utter unconsciousness, the specific gravity was high, averaging both in the *grey* and *white* matter 1041. He makes two exceptions in regard to the white matter, in both of which the specific gravity was below the mean; these were both cases of children of eight and ten years of age, and both of tubercular meningitis. Deducting these cases, the average specific gravity of the white matter in his series of cases, complicated with cerebral symptoms, was 1043.

On examining my cases in detail, I find that in most of those cases where the specific gravity of the grey matter was considerably below the mean, the patients had died of phthisis, and in other instances of exhaustion occurring at an advanced age. Where exceptions to this general inference were met with, it was found that either the symptoms immediately preceding death were of a grave character, or that the morbid appearance found in the membrane indicated chronic inflammatory action.

I have also taken the specific gravity of the grey and white matter of the cerebellum in forty-three cases. I have placed on the table the averages at different ages. The number of observations is too few to warrant more than a presumption that the specific gravity of the cerebellum is higher than that of the cerebrum. The difference is much more apparent when, instead of comparing averages, the specific gravity of the cerebellum is compared with that of the cerebrum to which it belonged.

I have not been able as yet to obtain a sufficient number of data showing the specific gravity of the cerebellum in the sane to furnish comparisons with the observations I have made. Dr. Sankey has kindly furnished me with a number of observations made by him; but as he has not separated the grey from the white matter, but has taken his specific gravity from a portion of brain containing both, I cannot compare my observations with his directly. I may state, however, that they tend to the same general conclusion, that the specific gravity of the cerebellum is higher than that of the cerebrum, and that it is so in healthy brains as well as in diseased ones, although probably in a smaller ratio.

Through the kindness of Dr. Haldane, I have been enabled to make a few observations on the specific gravity of the grey and white matter of the cerebellum in persons dying in the Royal Infirmary. The number of cases suitable for a comparison of this kind of cases dying without any cerebral symptoms, has as yet been rather too small to merit a special record. I may, however, state that in a series of five cases of males dying without cerebral symptoms, the average specific gravity of the grey substances of the cerebellum was 1042, that of the cerebrum being 1041. In the cases of the insane examined by me, the average was 1040 for the cerebellum, and 1035 only for the cerebrum, showing a much greater increase in the ratio in the insane than in the sane, although the absolute specific gravity was less in these particular cases.

The white substance of the cerebellum in the five cases referred to, had an average specific gravity of 1·0443, while that of the cerebrum was 1·0430. In the cases examined in the Asylum, the average specific gravity of the white substance of the cerebellum was 1·044, while that of the white substance of the cerebrum in the same cases was 1·039 only—thus showing a small increase in the absolute specific gravity of the white substance of the cerebellum in the insane in the cases compared, and a very considerable increase in the relative specific gravity to that of the cerebrum in the case of the insane.

From these data, although limited, I infer that the specific gravity of the cerebellum is increased in insanity, and attains a greater increase in relation to that of the cerebrum than it does in persons dying sane.

In comparing the specific gravity in the different forms of mental disease, and taking the average of all the cases of each kind, I find the lowest specific gravity of the grey matter to occur in cases of dementia, where, however, it is still 0·003 above the average in the sane. The next highest specific gravity occurs in cases of melancholy and monomania, the next in general paralysis, the next in mania, and the highest in epilepsy.

Of the white substance, the lowest average of specific gravity occurred in cases of mania, 1040; the next in dementia, 1041; the next highest in general paralysis, the next in monomania, 1044; and the highest also in epilepsy, being 1·0458.

The results of the preceding observations are curious and interesting. They are inferences certainly from a comparatively limited number of data; but they are, I think, sufficient to prove that more extended observations of a similar kind may probably lead to some satisfactory and important deductions regarding the pathology of insanity and the functions of the brain.

In conclusion, I think it right to state, that the specific gravity of the central substance was taken exactly in the same manner as that followed by Dr. Sankey. A number of jars were prepared with solutions of common salt, the density of which was determined by the urinometer. A series was prepared, ranging from 1·030 up to 1·050. Small portions of cerebral matter were dropped into these solutions, until a jar was found in which the portion so dropped floated midway, at the point of which it sank. This fluid gave the specific gravity, and to insure accuracy, and avoid fallacies arising from the spontaneous evaporation of the fluid, the specific of the fluid was in every experiment tested afresh by the urinometer at the time of the observation. The temperature of the room was 60° F. It is right to repeat the precaution pointed out by Dr. Sankey, namely, to take the first effect of the experiment, and that only; as by allowing the portion of brain to remain a few minutes only in the solution, its specific gravity rapidly alters by endosmose, and it will soon sink even in the strongest solutions. From not attending to this precaution, it is, I presume, to be explained why, in the pathological report lately published of a Metropolitan Asylum, the brain is stated in many cases to have had a specific gravity of 1·090 and upwards!

---

